# Clinical and Patient-Focused Outcomes After Percutaneous Screw Fixation of Pelvic Ring Fractures in Older Adults

**DOI:** 10.3390/jcm14113919

**Published:** 2025-06-03

**Authors:** Anna H. M. Mennen, Jan Boon, Jens A. Halm, Rolf W. Peters, Frank W. Bloemers, Daphne Van Embden

**Affiliations:** 1 Department of Surgery, Amsterdam UMC Location Academic Medical Centre, Meibergdreef 9, 1105 AZ Amsterdam, The Netherlands; 2 Department of Surgery, Amsterdam UMC Location Vrije Universiteit, De Boelelaan 1117, 1081 HV Amsterdam, The Netherlands

**Keywords:** percutaneous screw fixation, minimally invasive surgery, pelvic fracture, elderly, mobility

## Abstract

**Background:** Percutaneous screw fixation has increasingly been used for pelvic ring fracture fixation. In older adult patients, especially in patients with a fragility fracture of the pelvis (FFP), minimally invasive techniques followed by early ambulation have been promoted in order to regain pre-injury levels of mobility and independence. The purpose of this study was to evaluate patient-centered outcomes, including post-operative pain relief, functional performance, and satisfaction, in older adults with pelvic ring fractures treated with percutaneous screw fixation and to assess injury characteristics, complications, and return-to-home rates. **Methods:** In this retrospective cohort study, patients 50 years and older who had their pelvic fracture treated in the Amsterdam UMC location AMC between January 2019 and December 2022 were identified. After a minimum follow-up period of 6 months, a questionnaire was conducted by phone to evaluate the pain, current living situation, and mobility of the patients. **Results:** A total of 51 patients were included in this study with a median age of 74 years (IQR 62–82), and the vast majority were female (n = 40, 78%). Over half of the pelvic fractures were caused by low-energy trauma (n = 29, 57%). Unilateral or bilateral sacral fractures with unilateral anterior ring fractures were the most common fracture pattern. The interoperative complication rate was 4%, and the in-hospital complication rate was 23%. Forty-five patients were reached to complete the questionnaire. Forty patients (91%) returned to an acceptable level of mobility after treatment, and almost all (n = 44, 98%) were pleased with the results of the surgery. **Conclusions:** Percutaneous screw fixation of pelvic fractures in older adult patients is a safe and effective operating technique. Most patients preserve their pre-morbid functionality and are able to return to their previous place of residence after admission. Furthermore, patients are almost unanimously very pleased with the results of the surgery despite some residual pain complaints.

## 1. Introduction

As the Dutch population is ageing, the new generation of older adults often has an active lifestyle, which puts them at risk for both high- and low-energy trauma-related injuries [1,2]. While incidence rates for hip fractures have decreased in the last decade, several European countries note a rapid increase in the incidence of pelvic fractures [3,4].

The treatment of pelvic fractures has evolved in the last years from invasive open surgical procedures to minimally invasive percutaneous techniques. Open reduction and internal fixation (ORIF) requires more extensive exposure and thus causes a high level of surgical soft tissue injury. This technique is associated with a higher risk of several intra- and post-operative complications, including haemorrhage, deep venous thrombosis, neurovascular injuries, and infection [5]. In older adult patients, the extent of tissue disruption caused by the chosen surgical technique may hinder their recovery and delay early mobilization [6,7]. In contrast, minimally invasive percutaneous techniques like pubic rami screws, ante or retrograde, SI screws, fully trans sacral screws or ‘LC2-screws’ seem to have become more and more popular [8]. The potential benefits of the percutaneous techniques include less blood loss and shorter operating times, which are likely to result in better outcomes compared with open fixation [9]. Also, ‘new’ techniques, such as 3D navigated screw placement, are becoming more and more popular among pelvic surgeons in order to limit blood loss and operating times in this fragile patient population [10].

The goal of surgical stabilization is analgesia and to regain the pre-injury level of mobility and independence by early weight-bearing. In clinical practice, many of these patients seem to benefit greatly as pain relief is immediate. Restoring mobility quickly is especially important in older adult patients because they are at risk for immobility-related complications and muscle loss, even after a few days of immobility. In addition, pelvic fragility fractures are a serious burden to our healthcare system. The current 1-year mortality is 10–27%, and 44–75% of patients are unable to return to their own homes after hospitalization and become institutionalized [11,12]. Even though it seems very beneficial to treat older adults with a pelvic fracture by percutaneous screw fixation, the indication for surgical fixation and timing of the procedure is still very much a subject of debate.

The main purpose of this study was to describe patient-focused outcomes in terms of post-operative pain relief, functional performance, and patient satisfaction in a cohort of older adult patients with pelvic ring fractures after both high- and low-energy trauma that were treated by percutaneous screw fixation. Second, we assess the patient and injury characteristics, complications, and return to home rate.

## 2. Methods

### 2.1. Study Design and Study Setting

This study is a single-center retrospective cohort study with prospectively collected data. All patients older than 50 years who suffered a pelvic ring fracture and were surgically treated in the Amsterdam UMC location Academic Medical Centre (AMC) between January 2019 and December 2022 were identified by searching the hospital’s electronic patient files. Amsterdam UMC is a level 1 trauma center and tertiary referral center for pelvic and acetabular fracture surgery.

Patients were eligible for inclusion if they had sustained a pelvic ring fracture after both low or high-energy trauma and were surgically treated by percutaneous fixation only (e.g., no patients with open reduction and internal fixation (ORIF) of the anterior ring and with SI-screws in the posterior ring). Data collection for this study began after a minimum follow-up of 6 months post-surgery. This period was chosen to ensure that patients had adequate time for recovery and could provide reliable information on pain, mobility, and overall functional outcomes.

The operations were performed by at least one of three experienced pelvic and acetabular fracture surgeons (JAH, RWP, DVE). Using the percutaneous technique is not limited by the extent of pre-operative displacement but rather based on the intraoperative ability to stabilize the fracture adequately through closed means, which was successful in all patients. If applicable, percutaneous fixation was done according to the 360-degree principle; all fractures of the anterior and posterior ring were stabilized, if technically possible and clinically significant. All patients were treated using either ante- or retro-grade pubic rami screws, SI-screws, fully trans sacral screws, iliac screws, or ‘LC2 screws’. Both 7.3/6.5 (Synthes) fully and partially treated cannulated screws of titanium and stainless steel were used.

This study was exempt from the Medical Research Involving Human Subjects Act (WMO) by the Medical Ethics Review Committee of the Academic Medical Center (Ref. No. W21_033#21.037). Informed consent was obtained from all participants prior to their inclusion in the study.

### 2.2. Outcome Measures and Data Collection

The primary outcome measure of this study was mobility during follow-up, measured by the Parker Mobility Score (PMS). Secondary outcome measures were the level of pain during follow-up at rest and during mobilization, patient satisfaction regarding the results of the surgery, and the return to home rate.

After a minimum follow-up period of 6 months, patients were contacted by phone to collect prospective data on pain, mobility, current place of residence, and patient satisfaction regarding the surgery. The data was collected using a questionnaire consisting of 7 questions that were conducted by phone (see Table 1). Because our study population consists of older adult patients, we aimed to keep the questionnaire as short and concise as possible, using simple yes or no questions for patient satisfaction and the numerical pain reporting system (NRS) to evaluate pain. The results of questions 3–5 were used to calculate the Parker Mobility Score (PMS).

All fracture patterns were classified by three independent reviewers (AHM, RWP, DVE). Both high-energy and low-energy fractures were classified using descriptive terms and according to the Young and Burgess classification [13]. All fractures caused by low-energy trauma were additionally classified using the Rommens classification and OF-Pelvic classification [8,14].

Data on patient characteristics, American Society of Anesthesiologists (ASA) classification, comorbidities, trauma mechanism, fracture patterns, concomitant injuries, details on the operation, inter- or post-operative complications, the date of admission, length of hospital stay, and follow-up visits were retrospectively collected from the electronic patient files. Additionally, follow-up imaging was reviewed for possible complications with the osteosyntheses material and to determine bone union and malunion.

If a patient passed away during follow-up, their general practitioner was contacted to track down the expected cause of death and to determine how long after the surgery the patient died.

### 2.3. Definition

The term ‘older adult’ is used throughout this manuscript, as it has become preferred in recent research and clinical discussions regarding ‘elderly.’ This term is considered more respectful and better reflects the diversity and potential of this age group. Although the definition of ‘older adult’ is debated, it is commonly set at 50 years and older in osteoporosis guidelines and hip fracture studies, highlighting age-related changes in bone density and fracture risk from midlife onward.

Because there is no validated tool to measure mobility specifically after a pelvic fracture, the Parker Mobility Score was used to measure mobility in this study. The Parker Mobility Score is a validated and reliable score that answers three questions, each valued from 0–3 points, and is commonly used in clinical practice to monitor the mobility of older adult patients. A score of 0–3 is considered low, 4–6 moderate, and 7–9 reflects good mobility. The pre-injury and follow-up Parker Mobility Score was measured by direct inquiry.

In-hospital and follow-up pain levels were assessed using the Numerical Rating Scale (NRS), a validated instrument for pain measurement ranging from 0 to 10, where 0 represents no pain and 10 indicates the worst pain imaginable. In-hospital pain scores were retrospectively collected from the electronic patient files. To assess follow-up pain levels, patients were asked to self-report their pain levels at rest and during mobilization, providing a numerical score on the NRS scale.

Patient satisfaction was measured by direct inquiry using a single-item question: ‘Are you pleased with the results of your surgery?’ Participants were asked to respond with a binary choice of ‘yes’ or ‘no’, which allowed for a straightforward evaluation of overall satisfaction levels among the study cohort. If the answer was ‘no’, the patient was asked what the reason was for not being satisfied.

If it was not specified if a fall from stairs was at ground level or from the top of the stairs, this trauma mechanism was defined as high-energy trauma.

Comorbidities were assessed using the Charlson Comorbidity Score, which is a validated method for quantifying the burden of comorbid conditions in individuals. Each comorbidity is assigned a specific weight, and the sum of these weights results in the total score. The higher the total score, the greater the burden of comorbidities and the increased risk of adverse outcomes.

The post-treatment level of mobility is categorized as ‘acceptable’ under the following conditions: For patients initially classified as ‘high mobility group’ with a pre-injury Parker Mobility Score (PMS) ranging from 9 to 6, a minimum PMS of 6 should be attained after treatment. For those in the ‘low mobility group’ with a pre-injury PMS of 5 or lower, the objective was to regain their pre-injury PMS. This classification is grounded in the concept that a greater loss in PMS corresponds to a potential loss in functional independence.

### 2.4. Data Analysis

Statistical analysis was performed using the Statistical Package for the Social Sciences (SPSS) version 28.0 (SPSS, Chicago, IL, USA). The normality of continuous data was assessed using the Shapiro–Wilk test, and the missing values were not imputed. Descriptive analysis was conducted for the entire group, reporting mean and standard deviation for parametric data or median and percentiles for non-parametric data. Given the size of the cohort, no further statistical analyses were performed.

## 3. Results

**Figure 1:**
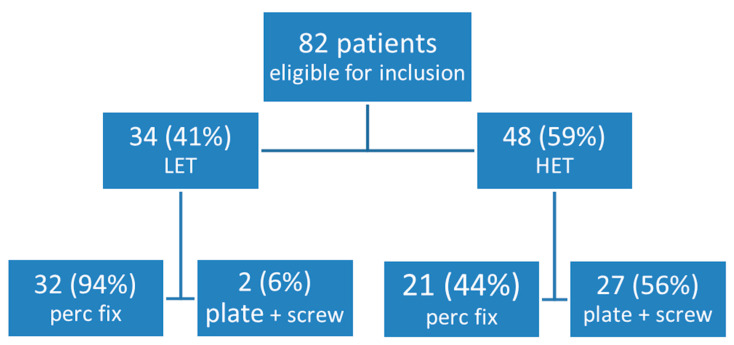
Type of trauma and type of surgery of the patients included in the study. HET, high-energy trauma; LET, low-energy trauma.

As shown in Figure 1, a total of 82 patients of 50 years or older with a pelvic ring fracture were identified, of whom 53 (65%) were treated fully by percutaneous fixation and 29 (35%) with open reduction and internal fixation (ORIF) of the anterior ring and with SI-screws for the posterior ring and were not part of the outcome analysis presented. Our reported outcomes focus solely on the percutaneous group. Of the 53 patients eligible for inclusion, one patient (2%) did not want to participate in this study, resulting in the inclusion of 52 patients.

The median age was 74 years old (IQR 62–82, range 50–90), and most patients were female (n = 40, 77%). The majority of the patients were either ASA class 2 (n = 21, 40%) or class 3 (n = 23, 44%). Only two patients (4%) were ASA class 1, and four (8%) were ASA class 4. The median Charlson Comorbidity Score was 4 (IQR 2–5).

Details of the descriptive, Young and Burgess, Rommens, and OF-PELVIC classification of the fracture patterns can be found in Table 2. Over half of the pelvic fractures were caused by low-energy trauma (n = 29, 56%). All patients had a closed pelvic fracture (n = 52, 100%). The most common descriptive fracture patterns were unilateral sacral fractures with unilateral anterior ring fractures (n = 16, 31%) or bilateral sacral fractures with unilateral anterior ring fractures (n = 10, 20%). Classifying the fracture patterns according to the Young and Burgess classification, LC1 fractures are the most common (n = 19, 37%). A total of 20 fractures could not be classified using the Young and Burgess classification, and 6 (30%) were caused by HET and 14 (70%) by LET. Approximately half of the LET pelvic fractures could not be classified using the Young and Burgess system (n = 14, 48%) and were thus grouped as ‘Other’. The LET pelvic fractures were additionally classified using the Rommens classification and the OF-Pelvic classification. The most common fracture pattern according to the Rommens classification was type IVb (n = 14, 48%), corresponding with OF-Pelvic classification type 4 (n = 14, 48%).

**Figure 2:**
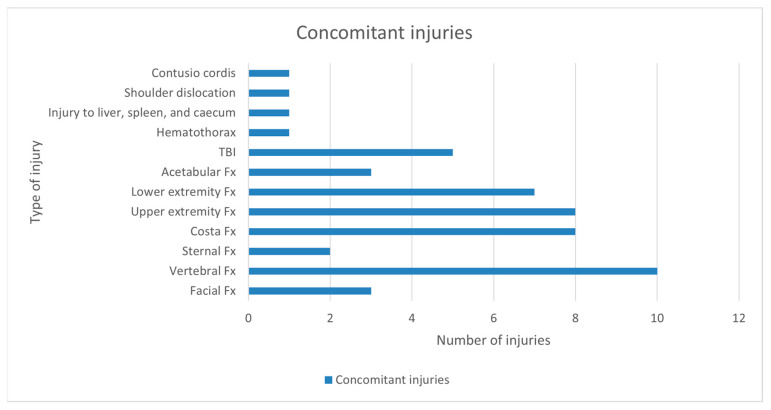
Type and frequency of concomitant injuries. Fx, fractures; TBI, traumatic brain injury.

Details of the type and frequency of the concomitant injuries are shown in Figure 2. Concomitant injuries were seen in 22 (43%) patients. The most common additional injuries were vertebral fractures (n = 10, 20%), upper extremity fractures (n = 8, 15%), and rib fractures (n = 8, 15%). Out of patients with pelvic ring fractures due to high-energy trauma, 73% (n = 16) had concomitant injuries. For patients with pelvic ring fractures due to low-energy trauma, 21% (n = 6) had concomitant injuries.

### 3.1. Operative Data, Direct Post-Operative Complications, and Follow-Up Imaging

Thirty patients (58%) were referred to our hospital for surgical fixation, and the median time to referral was 3 days (IQR 1–10, range 0–60 days).

Of all the patients who were surgically treated, 36 (69%) were surgically fixated within 2 weeks of sustaining the pelvic fracture and 16 (31%) after 2 weeks. All patients who were fixated within 2 weeks were in pain during the pelvic examination and unable to mobilize out of bed or walk 10 steps (n = 36, 100%). In addition, 23 (64%) had an unstable fracture pattern on initial imaging. Unstable fracture patterns were defined as fracture patterns more severe than the Young and Burgess type LC1. All the patients who were surgically treated after two weeks (n = 16, 31%) were not able to walk 10 steps without pain. Nine patients (56%) had an unstable fracture pattern on initial imaging.

Table 3 shows an overview of the surgical techniques per fracture pattern and weight-bearing regime. A total of 39 patients (76%) were treated according to the 360-degree principle, both anterior and posterior fixations. Patients who were not treated according to the 360-degree principle had either isolated sacral fractures with no anterior ring involvement or stabilization of the anterior fractures was not deemed clinically relevant. Of the 10 patients with anterior and posterior fractures who were not treated according to the 360-degree principle, 7 patients (70%) had a patient or doctor’s delay of at least 3 weeks (range 3–24 weeks) and the inability to mobilize was the main indication for operation.

All sacral fractures were stabilized using trans sacral trans iliacal sacral screws (TITSs), if technical possible, otherwise conventional sacral iliacal (SI) screws with the exception of 1 patient who presented very late after trauma (>6 months) with complaints of pain in the left groin and on imaging only delayed union of the left ramus fracture and sacral fractures in the process of fracture healing. In none of the patients was the percutaneous technique converted to open plate fixation or screw fixation.

Example case: An 86-year-old female sustained a left iliac wing fracture and superior and inferior pubic rami fractures after falling down stairs. The injury was classified as a Young–Burgess LC2 or Rommens FFP3a pattern (see Video S1 in the Supplementary Materials). Percutaneous fixation was performed using a pubic ramus screw and an LC2-screw under fluoroscopic guidance (see Video S2 and Figures S1–3 in the Supplementary Materials). At seven weeks post-operatively, the patient reported no pain and was ambulating independently without the need for assistive devices (see Figures S4 and S5 in the Supplementary Materials).

Pain scores pre-operative, 1 day post-operative, and at discharge were evaluated both at rest and during mobilization. The mean pain scores at rest and mobilization pre-operative were 4 (SD 2.6) and 6 (SD 2), respectively. One day post-operative, the mean pain score at rest was 3 (SD 1.8) and when mobilizing, 4 (SD 1.8). At discharge, the mean pain score at rest decreased to 2 (SD 1.5) and when mobilizing, 3 (SD 2.1).

The post-operative weight-bearing regime was most often weight-bearing as tolerated (WBAT), namely in 41 patients (80%). Of the patients who sustained a pelvic ring fracture after LET, 97% (n = 28) were allowed to immediately weight bear as tolerated, and of the pelvic ring fractures after HET, 61% (n = 13).

There were only two (4%) intraoperative complications, namely one patient who suffered from reversible cardiac ischemia without clinical impact due to significant hypotension caused by hypovolemia and another patient who was severely osteoporotic and suffered from iatrogenic fracture of the iliac wing.

A total of 17 in-hospital complications were recorded in 12 patients (23%). The most common complications were electrolyte disorders (n = 3, 18%), delirious episodes (n = 3, 18%), and urinary tract infections (n = 3, 18%). Additionally, there was one patient with acute kidney failure, one with decompensated liver cirrhosis, one wound infection, one osteomyelitis of the pubic bone, one pseudoaneurysm of the gluteal artery treated by thrombin injection, and one patient with severe hypotension and electrolyte imbalance caused by SIADH and hyperglycemia.

One patient (2%) was re-admitted within 30 days because of a strangulated ileus, most likely caused by a giant abdominal hernia, which was treated non-operatively. One patient (2%) had a second high-energy trauma leading to pelvic ring fractures within two months of the first trauma. After the initial trauma, he had percutaneous SI-screw fixation on one side, and after the second trauma, the new fractures on the contralateral side and both rami were percutaneously fixed.

There were two (4%) in-hospital deaths: a 90-year-old male who experienced pulmonary distress six days after surgery with a preexisting pulmonary restrictive disease and an 82-year-old female who had a cardiac arrest and passed away one day after surgery. Additionally, there were two (4%) out-of-hospital deaths: one 21 days after surgery due to COVID-19 in a 77-year-old female living in a nursing home and another three months after surgery in an 84-year-old female due to unknown causes. This resulted in a 1-year mortality rate of 8%.

The median length of hospital stay was 9 days (IQR 5–14). The median time between trauma and surgical fixation was 6 days (IQR 3–31) in the overall group. In the LET group, the median time between trauma and surgery was 6 days (IQR 4–28, range 1–266 days). The median time between hospital admission and surgery in the overall group was 2.5 days (IQR 1–4).

Of 42 patients (81%), follow-up imaging was available. Follow-up imaging showed five patients (12%) with backing out of their pubic and/or SI-screw, but none complained of pain. Two patients (5%) had breakage of their pubic screws (both fully threaded) without any pain complaints and fracture healing on imaging, and one patient (2%) of their TITS without any complaints.

Overall, six patients (10%) complained of pain, all located in the groin area. Five (10%) patients subsequently had their hardware removed; three (6%) of these patients were because of a low-grade infection; one patient had osteogenesis imperfecta, resulting in very brittle bones and limited grip for the pubic screw, which caused pain complaints; and one patient had persistent complaints of pain in the groin that could not be explained by osteosynthesis complications or an infection, and chose to have their hardware removed due to pain complaints. In addition, there was one patient who had a secondary dislocation of the ramus fracture, which caused the pain.

### 3.2. Patient-Focused Outcomes

The questionnaire was completed by 45 patients: 6 patients died before completing the questionnaire, resulting in a loss to follow-up of the questionnaire of 11.5%.

Most patients (n = 32, 71%) were able to mobilize without aids pre-injury (PMS 9), and 9% (n = 4) mobilized without aids indoors and with a walker outdoors (PMS 7). One patient was wheelchair-bound pre-injury (2%). At the time the questionnaire was conducted, 51% (n = 23) were able to mobilize without aids, and 16% were able to mobilize without aids indoors and with a walker outdoors (n = 7). In total, 91% (n = 40) of all patients returned to an acceptable level of mobility after treatment.

Almost all patients (n = 43, 96%) had no or mild pain complaints at rest (NRS 0–4). The other two patients (4%) had moderate (NRS 5–7) pain complaints at rest. During mobilization, 35 patients (78%) had no or mild pain, 9 patients (20%) had moderate pain, and one patient (2%) had severe pain due to pain complaints related to an infection.

Before their injury, 96% (n = 43) of the patients were living independently at home, and at the time of the questionnaire, 91% (n = 41) were still living independently at home. Only two patients (6%) who were previously living at home were now admitted to a nursing home.

When asked, almost all patients (n = 44, 98%) stated that they were pleased with the results of the surgery. The patient who was not satisfied complained of pain related to an infection.

## 4. Discussion

In this study, we present a detailed description of the patient and fracture characteristics in a cohort of older adult patients with pelvic fractures both after high- and low-energy trauma. Based on the results of this study, percutaneous fixation of pelvic fractures in older adult patients is a safe and effective technique for high- and low-energy fracture patterns. The minimally invasive technique, in combination with early weight bearing, seems to result in long-term functional independence.

Currently, studies in older adult patients with pelvic fractures mainly focus on discrete outcomes such as post-operative complications and mortality. In contrast, older adults often value quality over quantity of life years, with functional independence being one of their many priorities [15,16]. Seventy-four percent of older adult patients with life-threatening illnesses would even decline life-saving interventions if the intervention resulted in significant functional impairment [17]. In addition, most older adult patients wish to grow old in their own homes, so-called “aging in place” [18]. Aging in place is preferable not only from a patient’s perspective but also from a societal perspective. The relative costs are in favor of home-based care compared with institutional care [19]. In our cohort of patients, we found very favorable outcomes; at the time of the questionnaire, 91% of the patients had an acceptable level of mobility, and 96% were able to return to their previous place of residence after admission.

The outcome of older adult patients with pelvic ring injury is generally poor, with age being an independent predictor of mortality in patients with a pelvic fracture [20]. Morris et al. describe a 1-year mortality rate of 27% in a cohort of conservatively treated older adult patients with a pelvic fracture after both high- and low-energy trauma [21]. Of the surviving patients, only 58% were living independently at home at the end of their follow-up period. These results differ greatly from our cohort of percutaneous fixated patients, where the 1-year mortality rate was 8%, and 91% were living independently at home after 6 months.

While the results of our study show favorable outcomes regarding mobility, a direct comparison with open reduction and internal fixation (ORIF) is difficult. Most studies reporting on ORIF include younger adult patients, making comparison to our study challenging because these patients inherently have different outcomes [22,23]. Additionally, we believe that using ORIF as a mode of fixation for older adult patients is increasingly outdated due to its invasive nature and associated morbidity. This statement is supported by the recent literature that suggests that percutaneous fixation results in less soft-tissue trauma, reduced bleeding, shorter operative times, less invasive stabilization of the anterior ring, and lower infection risk [8,24,25,26]. As a result, minimally invasive fixation is increasingly favored [27].

To date, no randomized controlled trial has evaluated the outcomes of ORIF versus percutaneous fixation, specifically in older adults. While future studies comparing these techniques could clarify their relative impact on short- and long-term mobility outcomes, the established benefits of percutaneous fixation, particularly in the older population, raise ethical concerns regarding the feasibility of conducting such trials.

In addition, percutaneous fixation has become more and more common as a treatment for high-energy fracture patterns [6,28]. However, in clinical practice, a wide variety of surgical techniques are used. The current study shows that, even in high-energy fracture patterns, percutaneous techniques provide enough stability for early weight-bearing and are a preferable option compared to any open technique in older adult patients due to the minimal soft-tissue disruption, which allows for a faster recovery.

Posterior pelvic ring fractures are accompanied by anterior fractures in the vast majority of older adult patients after both LET and HET. In fragility fractures of the pelvis (FFPs) after a low-energy trauma, this phenomenon is described in up to 97% of the patients [8,29]. This is in line with the results in our cohort; isolated posterior injuries were only seen in 2 patients (4%). Although the biomechanical stability of the pelvis depends mainly on the posterior ring, the anterior ring contributes to 30% of the stability, which is compromised when fractured [30]. For adequate reduction and stabilization, anterior fixation according to the 360-degree principle could be considered in combined posterior and anterior injuries. Although several articles comparing combined anterior-posterior ring fixation versus posterior ring or anterior ring alone show no superiority between the two groups, a recent article by Tucker et al. shows that combined anterior-posterior fixation was associated with less opioid use and safe discharge home [31,32,33,34]. In addition, Wang et al. found that combined anterior-posterior fixation resulted in a significant difference regarding full early weight-bearing compared with solitary anterior fixation [35]. Historically, this anterior fixation was often performed by single or double plating. This technique provides high stability but is associated with high rates of screw loosening (46%), resulting in revision osteosyntheses in 13% of the patients [36]. In our cohort, we see results that support this; only one patient (2%) suffered from surgical wound infection. In contrast to anterior plating, our cohort of percutaneous fixated pubic fractures only showed backing out of screws in 10% (n = 5) of the patients, and none experienced any pain. Only one patient needed revision osteosynthese, which was due to infection and not related to the backing out of the screws. As the healthcare needs of older adult patients continue to grow, it is crucial that research on pelvic fractures takes into account the specific needs and preferences of this group. Future studies should focus more on patient-centered outcomes, such as functional recovery, quality of life, and pain management, rather than solely on mortality. In addition, it is important to investigate the potential long-term consequences of suboptimal healing, such as changes in load distribution during walking, which could put excessive strain on the sacroiliac joint or lower lumbar vertebrae. This may lead to chronic inflammation or postural low back pain, affecting a patient’s long-term quality of life. While advancements in understanding pelvic injuries have improved prognoses over time, residual disability remains a concern, highlighting the need for optimized treatment approaches [37].

### Limitations and Strengths

The main strength of this study is the extensive information presented on patient characteristics, fracture patterns, and patient-center outcome measurements. Moreover, a very high follow-up rate is achieved, which ensures that the results are representative and reliable.

We acknowledge that there are some limitations to our study. The retrospective study design results in possible bias due to missing information. In addition, the outcomes of our cohort of percutaneously fixated pelvic fractures have not been compared to the outcomes of fractures fixated by open reduction and internal fixation (ORIF). There was also some variation in the timing of the questionnaire that was conducted, varying between 6 months and 1.5 years after surgery, which could potentially influence the outcomes on mobility and living environment.

A limitation of this study is that no formal treatment algorithm was applied, and we did not stratify patients by displacement or trauma mechanism. All patients aged 50 years and older were treated percutaneously according to our institutional protocol. As such, no specific guidance for selecting CRIF versus ORIF can be derived. However, our findings demonstrate that percutaneous fixation can be safely applied across a broad spectrum of pelvic fractures in older adults, suggesting that institutions currently reserving CRIF for a limited subset may consider expanding its use. Furthermore, there might be some selection bias because over half (n = 30, 58%) of the patients in this cohort were referred to our hospital for surgical fixation. In the Netherlands, there is still a commonly held belief that many older adult patients with pelvic fractures are not eligible for fixation because it would either not improve their outcome or be too invasive. It is possible that only the most severe fracture patterns were discussed with an expertise center for potential surgical fixation and could, therefore, skew the results of our study. However, despite this potential bias, the overall results are very favorable, suggesting that the selection process for surgical fixation may have, in contrast, positively influenced the study results.

## 5. Conclusions

Percutaneous fixation in older adult patients with a pelvic fracture is a safe and effective operating technique for both high- and low-energy trauma. There are few interoperative and post-operative complications, and the 1-year mortality rate in this cohort of older adults is low (8%). Older adult patients who sustained a pelvic ring fracture after LET had a median time between trauma and surgery of 15 days (IQR 5–86). Furthermore, up to 91% of the patients achieved acceptable levels of mobilization after treatment. Most of the patients (96%) were able to return to their previous place of residence. Patients are almost unanimously (n = 44, 98%) pleased with the results of the surgery despite some residual pain complaints.

## Figures and Tables

**Table 1:** Study questionnaire to report on pain, mobility, current living situation, and patient satisfaction during follow-up.

Questionnaire	
1. Do you experience pain related to your pelvic fracture at rest?	NRS 0–10
2. Do you experience pain related to your pelvic fracture when walking?	NRS 0–10
3. What is your current level of independence during mobilization in your home?	I can walk independently I use a walking aid I use a wheelchair I am not capable of moving or being moved, e.g., immobile
4. What is your current level of independence during mobilization outside?	I can walk independently I use a walking aid I use a wheelchair I am not capable of moving or being moved, e.g., immobile
5. What is your current level of independence when going shopping/visiting a restaurant/or visiting family?	I can walk independently I use a walking aid I use a wheelchair I am not capable of moving or being moved, e.g., immobile
6. Where do you live currently?	At home At a (geriatric) rehabilitation center At a nursing home or assisted living
7. Are you pleased with the results of the operation?	Yes No

**Table 2:** Details of the descriptive, Young and Burgess, Rommens, and OF-Pelvic classifications of the fracture patterns.

Pt	MOI	Descriptive	YB	Rommens	OF-Pelvic
1	HET	Sacrum bilateral + anterior ring unilateral	Other (LC1 + isolated sacral Fx)	NA	NA
2	LET	Sacrum bilateral + anterior ring bilateral	Other (LC1 bilateral)	4b	4
3	LET	Sacrum bilateral + anterior ring unilateral	Other (LC1 + isolated sacral Fx)	4b	4
4	HET	Sacrum bilateral	Other (Bilateral isolated sacral Fx)	NA	NA
5	LET	Ilium unilateral + anterior ring unilateral	LC2	3a	5
6	LET	Sacrum bilateral + anterior ring unilateral	Other (LC1 + isolated sacral Fx)	4b	4
7	LET	Sacrum bilateral + anterior ring unilateral	Other (LC1 + sacral Fx)	4b	4
8	HET	Sacrum unilateral + anterior ring unilateral	LC1	NA	NA
9	LET	Sacrum unilateral + anterior ring bilateral	LC1	2c	3
10	LET	Sacrum bilateral + anterior ring bilateral	Other (LC1 bilateral)	4b	4
11	HET	Sacrum unilateral + anterior ring unilateral	LC1	NA	NA
12	LET	Ilium unilateral + anterior ring unilateral	LC2	3a	5
13	HET	Ilium unilateral + anterior ring unilateral	LC2	NA	NA
14	LET	Ilium unilateral + anterior ring unilateral	LC2	3a	5
15	LET	Sacrum unilateral + anterior ring bilateral	LC1	2c	3
16	HET	Sacrum unilateral + anterior ring unilateral	LC1	NA	NA
17	LET	Sacrum bilateral + anterior ring unilateral	Other (LC1 + isolated sacral Fx)	4b	4
18	HET	Sacrum unilateral + anterior ring unilateral	LC1	NA	NA
19	HET	Si-diastase unilateral	Other (Isolated unilateral SI-diastase)	NA	NA
20	HET	Sacrum bilateral + ilium unilateral + anterior ring bilateral	Other (LC2 + LC1)	NA	NA
21	HET	Ilium unilateral + anterior ring unilateral	LC2	NA	NA
22	HET	Sacrum bilateral + anterior ring unilateral	Other (LC1 + isolated sacral Fx)	NA	NA
23	HET	Si-diastase bilateral + anterior ring bilateral	APC2	NA	NA
24	LET	Sacrum bilateral + anterior ring unilateral	Other (LC1 + isolated sacral Fx)	4b	4
25	LET	Sacrum bilateral + anterior ring unilateral	Other (LC1 + isolated sacral Fx)	4b	4
26	LET	Sacrum unilateral + anterior ring unilateral	LC1	2c	3
27	HET	Sacrum unilateral + anterior ring unilateral	LC1	NA	NA
28	LET	Sacrum unilateral + anterior ring unilateral	LC1	NA	NA
29	HET	Sacrum unilateral + ilium unilateral + anterior ring unilateral	LC2	NA	NA
30	HET	Ilium unilateral + anterior ring unilateral	LC2	NA	NA
31	HET	Sacrum unilateral + anterior ring bilateral	LC1	NA	NA
32	LET	Sacrum unilateral + anterior ring unilateral	LC1	NA	NA
33	HET	Sacrum unilateral + anterior ring unilateral	LC1	NA	NA
34	LET	Ilium unilateral + anterior ring unilateral	LC2	NA	NA
35	LET	Sacrum unilateral + anterior ring unilateral	LC1	NA	NA
36	LET	Sacrum unilateral + anterior ring unilateral	LC1	NA	NA
37	HET	Sacrum bilateral + ilium unilateral + anterior ring bilateral	Other (LC2 + LC1)	NA	NA
38	HET	Sacrum unilateral + anterior ring unilateral	LC1	NA	NA
39	LET	Sacrum bilateral + anterior ring unilateral	Other (LC1 + isolated sacral Fx)	4b	4
40	LET	Sacrum bilateral + anterior ring bilateral	Other (LC1 bilateral)	4b	4
41	HET	Sacrum unilateral + anterior ring bilateral	LC1	NA	NA
42	LET	Sacrum bilateral + anterior ring bilateral	Other (LC1 bilateral)	NA	NA
43	LET	Sacrum bilateral + anterior ring unilateral	Other (LC1 bilateral)	NA	NA
44	LET	Sacrum unilateral + anterior ring unilateral	LC1	NA	NA
45	HET	Sacrum unilateral + anterior ring unilateral	LC1	NA	NA
46	LET	Sacrum unilateral + anterior ring unilateral	LC1	NA	NA
47	LET	Ilium unilateral + anterior ring unilateral	LC2	3a	5
48	LET	Sacrum unilateral + anterior ring unilateral	LC2	2c	5
49	LET	Sacrum bilateral + anterior ring bilateral	Other (LC1 bilateral)	4b	4
50	HET	Sacrum unilateral + ilium unilateral + anterior ring unilateral	LC2	NA	NA
51	HET	Sacrum unilateral + anterior ring unilateral	LC1	NA	NA
52	LET	Sacrum bilateral + anterior ring bilateral	Other (LC1 bilateral)	NA	NA

HET, high-energy trauma; LET, low-energy trauma; MOI, mechanism of injury; NA, not applicable; Pt, patient.

**Table 3:** Details of surgical fixation per fracture pattern and post-operative weight-bearing regime.

Pt	MOI	Fracture Pattern	Surgical Fixation	Weight-Bearing Regime
1	HET	Sacrum bilateral + anterior ring unilateral	Bilateral 1x SI-screw	WBAT
2	LET	Sacrum bilateral + anterior ring bilateral	Bilateral 1x TITS	WBAT
3	LET	Sacrum bilateral + anterior ring unilateral	Bilateral 1x SI-screw	WBAT
4	HET	Sacrum bilateral	Bilateral 1x SI-screw	WBAT
5	LET	Ilium unilateral + anterior ring unilateral	Unilateral 1x LC2-screw + unilateral pubic screw	1 leg TTWB, 1 leg FWB 8w
6	LET	Sacrum bilateral + anterior ring unilateral	Unilateral pubic screw	WBAT
7	LET	Sacrum bilateral + anterior ring unilateral	Unilateral 1x TITS + unilateral pubic screw	WBAT
8	HET	Sacrum unilateral + anterior ring unilateral	Unilateral 1x TITS	WBAT
9	LET	Sacrum unilateral + anterior ring bilateral	Unilateral 2x TITS	WBAT
10	LET	Sacrum bilateral + anterior ring bilateral	Bilateral 1x TITS	WBAT
11	HET	Sacrum unilateral + anterior ring unilateral	Bilateral 1x SI-screw + unilateral pubic screw	WBAT
12	LET	Ilium unilateral + anterior ring unilateral	Unilateral 1x LC2-screw + unilateral pubic screw	WBAT
13	HET	Ilium unilateral + anterior ring unilateral	Unilateral 1x LC2-screw + unilateral pubic screw	1 leg TTWB, 1 leg FWB 8w
14	LET	Ilium unilateral + anterior ring unilateral	Unilateral 2x iliac screw + unilateral pubic screw	WBAT
15	LET	Sacrum unilateral + anterior ring bilateral	Unilateral 2x SI-screw + bilateral pubic screw	WBAT
16	HET	Sacrum unilateral + anterior ring unilateral	Bilateral 1x TITS + bilateral pubic screw	1 leg NWB, 1 leg FWB 8w
17	LET	Sacrum bilateral + anterior ring unilateral	Unilateral 1x TITS + unilateral pubic screw	WBAT
18	HET	Sacrum unilateral + anterior ring unilateral	Unilateral 2x SI-screw	WBAT
19	HET	Si-diastase unilateral	Unilateral 1x SI-screw	1 leg NWB, 1 leg FWB 8w
20	HET	Sacrum bilateral + ilium unilateral + anterior ring bilateral	Bilateral 1x SI-screw + bilateral pubic screw	Both legs NWB 6w
21	HET	Ilium unilateral + anterior ring unilateral	Unilateral 1x LC2-screw + unilateral pubic screw	Both legs NWB 6w
22	HET	Sacrum bilateral + anterior ring unilateral	Bilateral 1x SI-screw + unilateral pubic screw	Both legs NWB due to calcaneal Fx
23	HET	Si-diastase bilateral + anterior ring bilateral	Unilateral 1x TITS + contralateral 1x SI-screw + bilateral pubic screw	Both legs NWB 8w
24	LET	Sacrum bilateral + anterior ring unilateral	Unilateral 1x SI-screw + 1x TITS + unilateral pubic screw	WBAT
25	LET	Sacrum bilateral + anterior ring unilateral	Bilateral 1x TITS + unilateral pubic screw	WBAT
26	LET	Sacrum unilateral + anterior ring unilateral	Unilateral 1x TITS + unilateral pubic screw	WBAT
27	HET	Sacrum unilateral + anterior ring unilateral	Unilateral 1x TITS + 1x SI-screw + unilateral pubic screw	WBAT
28	LET	Sacrum unilateral + anterior ring unilateral	Unilateral 1x TITS + unilateral pubic screw	WBAT
29	HET	Sacrum unilateral + ilium unilateral + anterior ring unilateral	Unilateral 2x TITS + unilateral pubic screw	WBAT
30	HET	Ilium unilateral + anterior ring unilateral	Unilateral 1x LC2-screw + unilateral pubic screw	1 leg TTWB, 1 leg FWB 6w
31	HET	Sacrum unilateral + anterior ring bilateral	Unilateral 2x SI-screw + bilateral pubic screw	Both legs NWB 6w
32	LET	Sacrum unilateral + anterior ring unilateral	Unilateral 1x TITS	WBAT
33	HET	Sacrum unilateral + anterior ring unilateral	Unilateral 1x TITS + unilateral pubic screw	WBAT
34	LET	Ilium unilateral + anterior ring unilateral	Unilateral 1x TITS + 1x iliac screw + unilateral pubic screw	WBAT
35	LET	Sacrum unilateral + anterior ring unilateral	Unilateral 1x TITS + unilateral pubic screw	WBAT
36	LET	Sacrum unilateral + anterior ring unilateral	Unilateral 1x TITS + unilateral pubic screw	WBAT
37	HET	Sacrum bilateral + ilium unilateral + anterior ring bilateral	Bilateral 1x SI-screw + unilateral pubic screw	WBAT
38	HET	Sacrum unilateral + anterior ring unilateral	Unilateral 1x TITS + unilateral pubic screw	WBAT
39	LET	Sacrum bilateral + anterior ring unilateral	Unilateral 1x TITS + unilateral pubic screw	WBAT
40	LET	Sacrum bilateral + anterior ring bilateral	Unilateral 1x TITS + bilateral pubic screw	WBAT
41	HET	Sacrum unilateral + anterior ring bilateral	Unilateral 1x TITS + bilateral pubic screw	WBAT
42	LET	Sacrum bilateral + anterior ring bilateral	Unilateral 2x TITS + bilateral pubic screw	WBAT
43	LET	Sacrum bilateral + anterior ring unilateral	Bilateral 1x TITS + unilateral pubic screw	WBAT
44	LET	Sacrum unilateral + anterior ring unilateral	Unilateral 1x TITS	WBAT
45	HET	Sacrum unilateral + anterior ring unilateral	Unilateral 1x SI-screw + 1x TITS + unilateral pubic screw	WBAT
46	LET	Sacrum unilateral + anterior ring unilateral	Unilateral 1x TITS + unilateral pubic screw	WBAT
47	LET	Ilium unilateral + anterior ring unilateral	Unilateral 1x iliac screw + unilateral pubic screw	WBAT
48	LET	Sacrum unilateral + anterior ring unilateral	Unilateral 1x SI-screw + unilateral pubic screw	WBAT
49	LET	Sacrum bilateral + anterior ring bilateral	Unilateral 1x TITS + unilateral pubic screw	WBAT
50	HET	Sacrum unilateral + ilium unilateral + anterior ring unilateral	Unilateral 1x SI-screw + 1x TITS + unilateral pubic screw	WBAT
51	HET	Sacrum unilateral + anterior ring unilateral	Unilateral 2x TITS + unilateral pubic screw	WBAT
52	LET	Sacrum bilateral + anterior ring bilateral	Bilateral 1x TITS + bilateral pubic screw	WBAT

Fx, fracture; FWB, full weight bearing; HET, high-energy trauma; LET, low-energy trauma; MOI, mechanism of injury; NWB, non-weight bearing; TTWB, toe-touch weight bearing; WBAT, weight bearing as tolerated.

## Data Availability

The data presented in this study are available on request from the corresponding author.

## Supplementary Materials

The following supporting information can be downloaded at: https://www.mdpi.com/article/10.3390/jcm14113919/s1, Video S1 axial CT scan of the pelvis demonstrating the fracture pattern; Video S2 manipulation under anesthesia (MUA) of the LC2 fracture.
