# Supplementary material for: Clinical and Patient-Focused Outcomes After Percutaneous Screw Fixation of Pelvic Ring Fractures in Older Adults
Source: J Clin Med. 2025 Jun 3;14(11):3919. doi: 10.3390/jcm14113919 (PMC12155743; doi:10.3390/jcm14113919)
Supplement: Supplementary file 1 [file jcm-14-03919-s001.zip › jcm-3361297-supplementary.pdf]

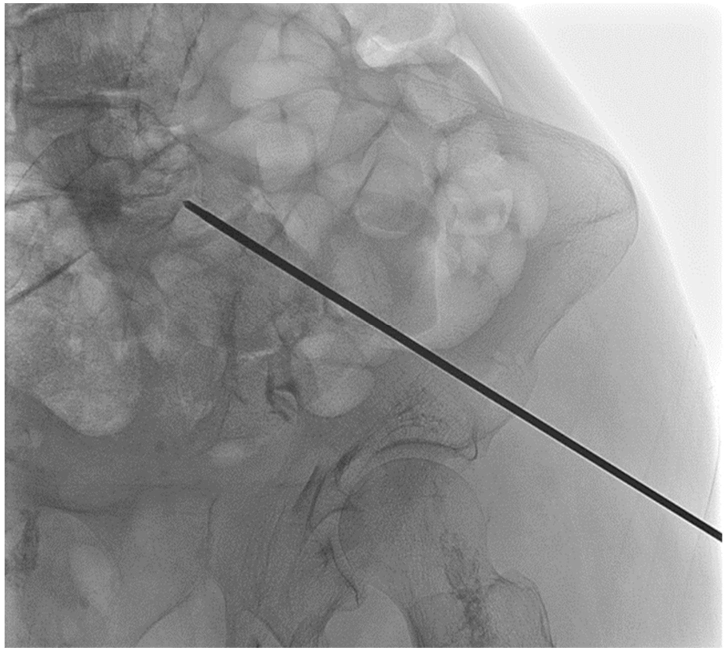

Figure S1. Shows intraoperative placement of the K-wire for the LC2 screw.

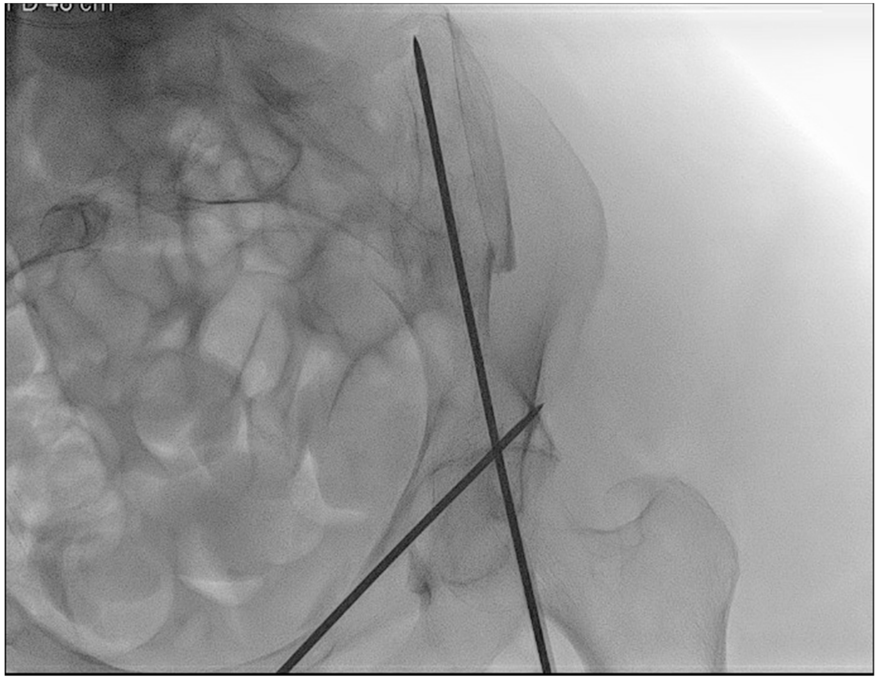

Figure S2. Demonstrates positioning of the K-wires for the LC2 and pubic screws during surgery.

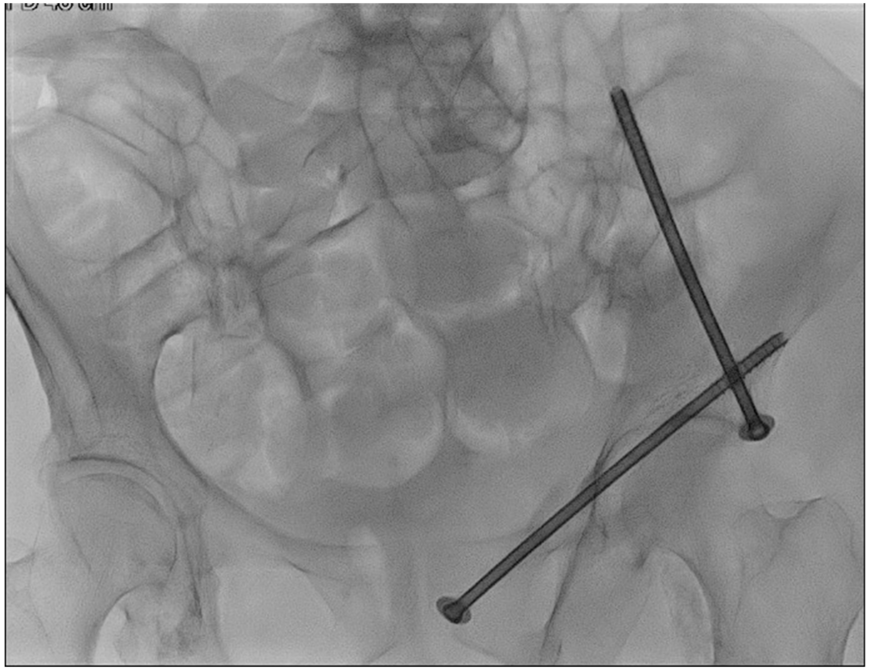

Figure S3. Shows intraoperative fluoroscopic imaging of the LC2 screw and pubic ramus screw placement.

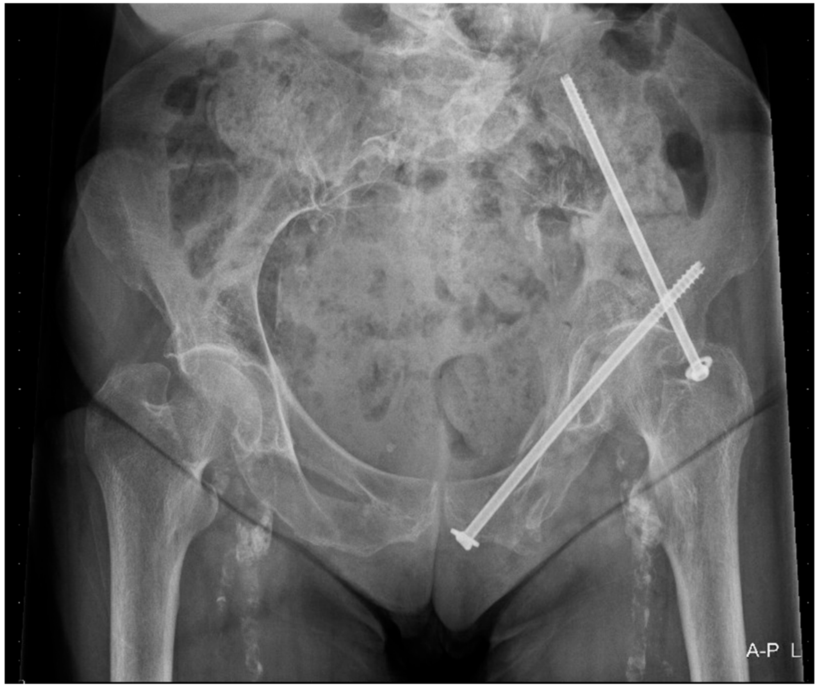

Figure S4. Shows inlet view imaging at 7 weeks postoperatively.

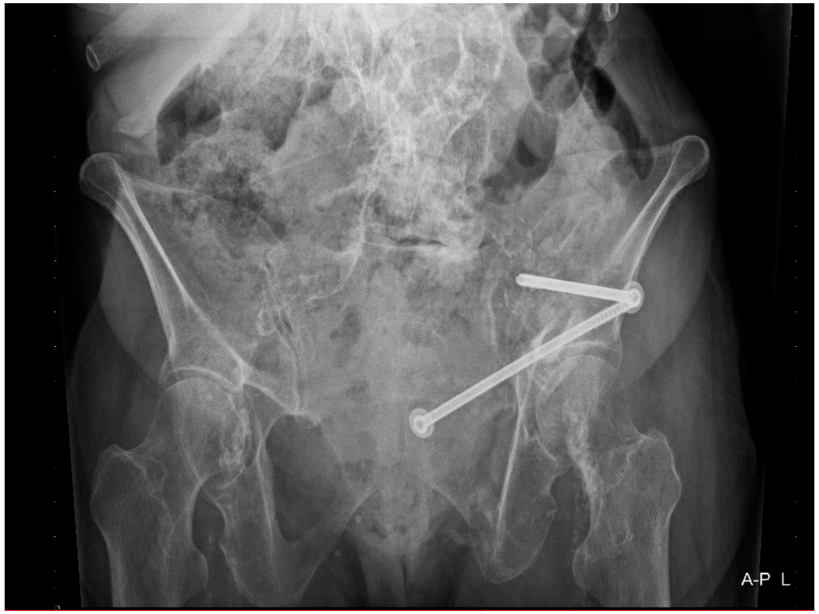

Figure S5. Shows outlet view imaging at 7 weeks postoperatively.

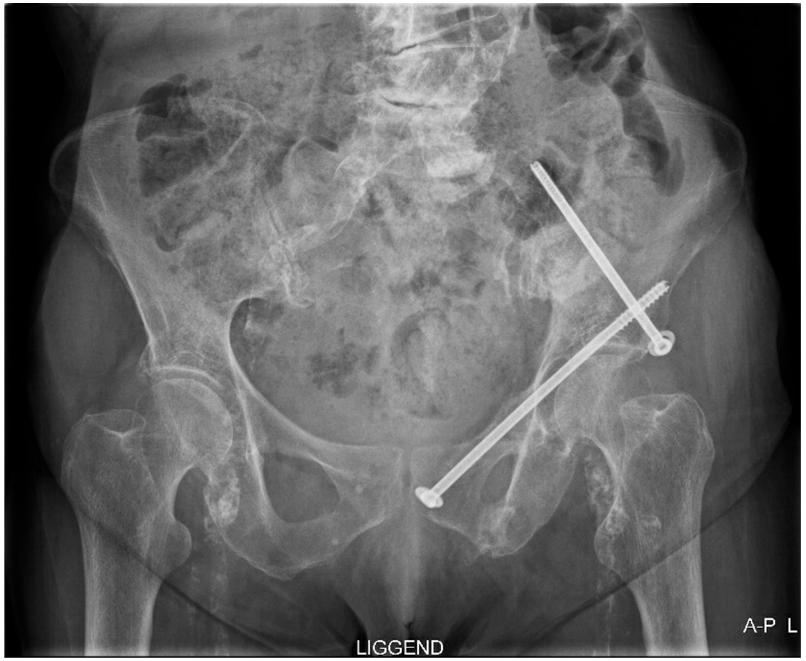

Figure S6. Shows AP view imaging at 7 weeks postoperatively.
